# Supplementary material for: Causal Relationship between Aging and Anorexia Nervosa: A White-Matter-Microstructure-Mediated Mendelian Randomization Analysis
Source: Biomedicines. 2024 Aug 16;12(8):1874. doi: 10.3390/biomedicines12081874 (PMC11351342; doi:10.3390/biomedicines12081874)
Supplement: Supplementary file 1 [file biomedicines-12-01874-s001.zip › Supplementary Figure/Figure S4/Singe SNP plot of MR analysis for TL to ubm-b-1452.pdf]

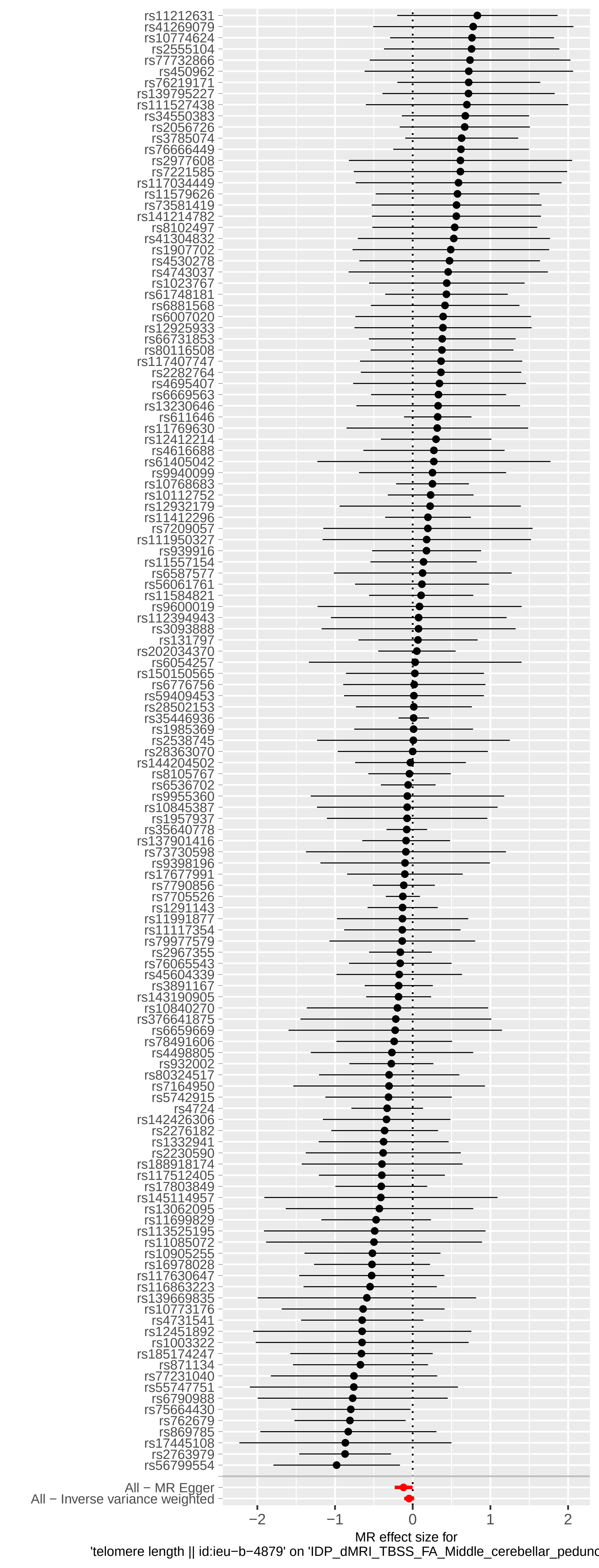

rs11212631
rs41269079
rs10774624
rs2555104
rs77732866
rs450962
rs76219171
rs139795227
rs111527438
rs34550383
rs2056726
rs3785074
rs76666449
rs2977608
rs7221585
rs117034449
rs11579626
rs73581419
rs141214782
rs8102497
rs41304832
rs1907702
rs4530278
rs4743037
rs1023767
rs61748181
rs6881568
rs6007020
rs12925933
rs66731853
rs80116508
rs117407747
rs2282764
rs4695407
rs6669563
rs13230646
rs611646
rs11769630
rs12412214
rs4616688
rs61405042
rs9940099
rs10768683
rs10112752
rs12932179
rs11412296
rs7209057
rs111950327
rs939916
rs11557154
rs6587577
rs56061761
rs11584821
rs9600019
rs112394943
rs3093888
rs131797
rs202034370
rs6054257
rs150150565
rs6776756
rs59409453
rs28502153
rs35446936
rs1985369
rs2538745
rs28363070
rs144204502
rs8105767
rs6536702
rs9955360
rs10845387
rs1957937
rs35640778
rs137901416
rs73730598
rs9398196
rs17677991
rs7790856
rs7705526
rs1291143
rs11991877
rs11117354
rs79977579
rs2967355
rs76065543
rs45604339
rs3891167
rs143190905
rs10840270
rs376641875
rs6659669
rs78491606
rs4498805
rs932002
rs80324517
rs7164950
rs5742915
rs4724
rs142426306
rs2276182
rs1332941
rs2230590
rs188918174
rs117512405
rs17803849
rs145114957
rs13062095
rs11699829
rs113525195
rs11085072
rs10905255
rs16978028
rs117630647
rs116863223
rs139669835
rs10773176
rs4731541
rs12451892
rs1003322
rs185174247
rs871134
rs77231040
rs55747751
rs6790988
rs75664430
rs762679
rs869785
rs17445108
rs2763979
rs56799554
All − MR Egger
All − Inverse variance weighted
−2
−1
0
1
2
MR effect size for
'telomere length || id:ieu−b−4879' on 'IDP_dMRI_TBSS_FA_Middle_cerebellar_peduncl
